# What aspects of pain and functional outcomes contribute to patient satisfaction 1 year after surgery within total hip and total knee arthroplasty populations? A registry-based cohort study

**DOI:** 10.2340/17453674.2026.45732

**Published:** 2026-05-05

**Authors:** Marys REVAZ, Thomas PERNEGER, Christophe BAREA, Hermes H MIOZZARI, Didier HANNOUCHE, Anne LÜBBEKE

**Affiliations:** 1 Division of Orthopedic Surgery and Musculoskeletal Trauma Care, Geneva University Hospitals, Faculty of Medicine, University of Geneva, Geneva, Switzerland; 2 Division of Clinical Epidemiology, Geneva University Hospitals, Geneva, Switzerland; 3 Nuffield Department of Orthopaedics, Rheumatology and Musculoskeletal Sciences, University of Oxford, UK; ORCIDs, if available, can be found on the article page (https://www.actaorthop.org/actao/)

## Abstract

**Background and purpose:**

Patient satisfaction after total hip and knee arthroplasty (THA, TKA) is influenced by multiple factors, including patient-reported pain and function. We aimed to examine whether functional abilities or pain during specific activities are associated more than other aspects with satisfaction 1 year after THA and TKA.

**Methods:**

This cohort study included all primary elective THAs and TKAs performed between January 2012 and June 2022 at a tertiary care university hospital. Ordinal logistic regression models were used to assess associations between patient satisfaction and WOMAC Pain and Function summary scores and items, 1 year postoperatively.

**Results:**

1,772 THAs and 1,323 TKAs were included. Individually, all scores and items were associated with satisfaction. However, multivariable models revealed differences in the strength of association. Global pain score (odds ratio [OR] THA: 2.80, 95% confidence interval [CI] 2.40–3.29; TKA: 3.61, CI 2.96–4.44), pain while walking on the flat (OR THA: 1.79, CI 1.45–2.22; TKA: 1.74, CI 1.43–2.11), and pain going up or down stairs (OR THA: 1.64, CI 1.37–1.96; TKA: 1.68, CI 1.44–1.97) showed the strongest associations in both populations. Among THAs, activities walking on the flat (OR 1.41, CI 1.17–1.68), putting on socks (OR 1.29, CI 1.12–1.48), and ascending stairs (OR 1.26, CI 1.08–1.46) contributed most to satisfaction, whereas among TKAs, walking on the flat (OR 1.41, CI 1.18–1.69), rising from sitting (OR 1.32, CI 1.11–1.58), and getting in or out of a car (OR 1.31, CI 1.10–1.57) did.

**Conclusion:**

After THA/TKA, patient satisfaction is associated with pain during basic daily tasks—especially walking and stair climbing. Key functional drivers differ by joint: socks and stairs matter for THA, rising from sitting and getting in/out of a car matter for TKA.

Patient satisfaction is a multidimensional construct comprising 2 main aspects: satisfaction with the process of care and with its outcomes [1]. In the literature, satisfaction with the outcomes of total hip and knee arthroplasty (THA, TKA) has been related to many factors, including the concept of embodiment, patient expectations, and patient-reported outcomes (PROs). Embodiment refers to the relationship between the body and the self, individuals typically perceiving them as one. Illness or injury can disrupt this unity, causing the altered body part to feel disconnected from the self. The inability to achieve re-embodiment, i.e., integrate the prosthesis into the body, has been linked to dissatisfaction [2-4]. Patient satisfaction has also been associated with preoperative patient expectations, with unfulfilled expectations contributing to dissatisfaction. In addition, it has been linked to PROs, particularly improvements in pain, function, and general health-related quality of life, as well as postoperative pain, functional abilities and preoperative mental health [5-7].

While most studies have examined the relationship between overall patient expectations or PRO scores and satisfaction, only a few studies have explored how specific aspects of these expectations or outcomes relate to patient satisfaction. These studies have identified that certain patient-reported functional abilities, such as climbing up or down stairs after TKA, and the fulfillment of specific expectations, such as improved knee range of motion (ROM) after TKA, have a greater influence on patient satisfaction than others [8-11]. However, the literature on this topic remains limited, focusing primarily on TKA patients, with pain experienced during specific activities yet to be explored.

Therefore, we aimed to determine whether, and which, functional abilities or pain experienced while performing specific activities are more strongly associated with patient satisfaction than others 1 year after THA and TKA.

## Methods

### Study design and population

For this cohort study, all primary elective THAs and TKAs performed between January 2012 and June 2022 at a tertiary care university hospital, and collected as part of the Geneva Arthroplasty Registry [12], were eligible. This institutional registry founded in 1996 has 100% completeness of primary elective THA and TKA registration [13]. Arthroplasties were excluded if patients had not consented to research, had died, were lost to follow-up, or had undergone revision within 1 year after surgery, as well as those with an incomplete or missing short-form (12-item scale) of the Western Ontario and McMasters Universities Osteoarthritis Index (WOMAC) [14] or missing patient-reported satisfaction at 1-year follow-up. This study adheres to STROBE guidelines.

### Study variables

The main study variables included patient satisfaction, as well as WOMAC Pain and Function summary scores and items 1 year after surgery. Patient satisfaction was assessed with the following question: “Are you satisfied with the result of your hip/knee surgery?” on a 5-point Likert scale: (1) dissatisfied, (2) somewhat dissatisfied, (3) somewhat satisfied, (4) satisfied, and (5) very satisfied. WOMAC scores and items were assessed with the short-form WOMAC questionnaire, designed specifically for patients with hip or knee osteoarthritis, and comprising 12 items, 5 for pain (pain while walking on the flat, going up or down stairs, at night while in bed, rising from or sitting on chair, and standing upright) and 7 for function (difficulty in ascending stairs, rising from sitting, walking on the flat, getting in or out of car, putting on socks, rising from bed, and sitting). Each item is rated on a 5-point Likert scale, and both pain and function summary scores range from 0 to 100, with higher scores indicating better outcomes [14]. In this study, only WOMAC scores and items at 1-year follow-up were investigated; changes in outcomes from baseline to 1 year were not analyzed. Indeed, preliminary analyses presented in the Supplementary Table (see Appendix) indicated that patient satisfaction is more strongly influenced by postoperative scores than by changes in scores. This finding is consistent with previous literature, which emphasizes the greater importance of postoperative scores over changes in scores for patient satisfaction [15,16].

Descriptive variables assessed preoperatively included sex, age at the time of surgery, body mass index (BMI), American Society of Anesthesiologists Physical Status (ASA) score [17], number of comorbidities, smoking status (never, former or current), education level (≤ 8 years, 9–12 years or ≥ 13 years), insurance type (public or private), diagnosis (primary or secondary osteoarthritis), University of California Los Angeles (UCLA) activity scale [18], Mental Component Score (MCS) and Physical Component Score (PCS) of the 12-item Short Form (SF-12) questionnaire [19], self-rated health (SF-12 first item), and pain and function summary scores of the short-form WOMAC questionnaire [14]. The ASA score assesses a patient’s pre-anesthesia comorbidity status (1: normal healthy patient, 2: patient with mild systemic disease, 3: patient with severe systemic disease, and 4: patient with severe systemic disease that is a constant threat to life) [17]. The UCLA activity scale assesses a patient’s activity level on a scale from 1 (wholly inactive) to 10 (regularly participates in impact sports) (18), and was categorized into low (score 1–4), moderate (score 5–7), and high activity (score 8–10). The SF-12 questionnaire assesses a patient’s general health status and comprises 2 summary scores, MCS and PCS [19].

### Statistics

All analyses were performed separately for the THA and TKA populations. Baseline characteristics were reported separately for each population, with continuous variables reported as mean and standard deviation (SD) and categorical variables as proportions (in %). Mean WOMAC Pain and Function summary scores at 1-year follow-up were calculated for each satisfaction level to compare their association with patient satisfaction in both populations. To evaluate and compare the contributions of WOMAC Pain and Function summary scores as well as individual items to patient satisfaction, univariable and multivariable ordinal logistic regression models were performed. Patient satisfaction, coded from 1 to 5 (= best), was used in all models as the response variable. In univariable models, WOMAC Pain summary score, WOMAC Pain individual items (5 items), WOMAC Function summary score, and WOMAC Function individual items (7 items) were alternately used as the explanatory variable. In multivariable models, 1 model including both summary scores, 1 including all pain items, and another including all function items were constructed. The WOMAC individual items were coded from 1 to 5 and were evaluated as continuous variables. A total of 14 univariable and 3 multivariable models were built for each population. Given the exploratory nature of the analyses, no multiplicity adjustment was applied, and odds ratios (ORs) with 95% confidence intervals (CIs) were reported. They were reported for a difference of 1 SD for summary scores and for 1 point on the response scale for items. In ordinal logistic regression, an OR is a measure of the relative change in odds of observing a higher vs lower category in the ordinal outcome for a unit increase in a predictor variable while holding other predictors in the model constant [20]. All analyses were conducted using a complete-case approach. Data were analyzed using R, version 4.2.2 (R Foundation for Statistical Computing, Vienna, Austria).

### Ethics, data sharing, funding, and disclosures

Written informed consent was obtained from all patients included in this study, and ethical approval was granted by the local ethics committee (CCER Geneva, Switzerland). The data supporting this study is not publicly available due to privacy or ethical restrictions but is available from the corresponding author upon reasonable request. Institutional financial support for the registry was provided by the “Fondation pour la recherche ostéoarticulaire.” The authors declare no conflicts of interest. Complete disclosure of interest forms according to ICMJE are available on the article page, doi: 10.2340/17453674.2026.45732

## Results

**Figure 1:**
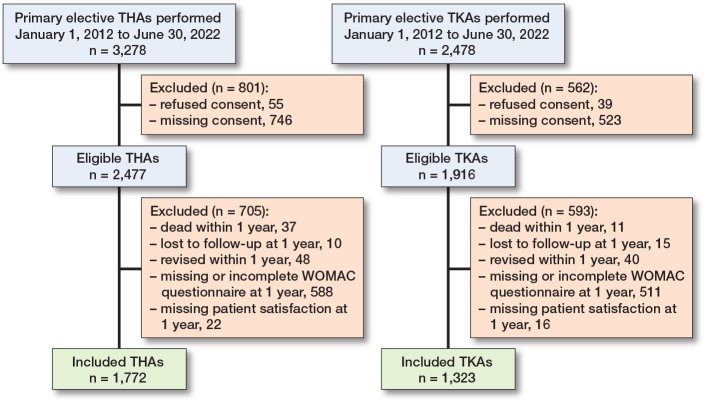
Flowcharts for total hip arthroplasty (THA) and total knee arthroplasty (TKA) populations.

Between January 2012 and June 2022, 3,278 primary elective THAs and 2,478 primary TKAs were performed. Of those, 2,477 THAs and 1,916 TKAs met the eligibility criteria (i.e., patients gave consent to research), and 1,772 THAs and 1,323 TKAs were included in the analysis, including only procedures with complete WOMAC questionnaire and patient satisfaction question at 1-year follow-up (Figure 1).

Preoperatively, patients with THA were less often female (55% vs 64%), were slightly younger (68.8 vs 70.9 years), and had lower BMI (26.9 vs 29.7) than patients with TKA. They generally had lower ASA grades and fewer comorbidities, were more often smokers and had higher education levels. Activity levels, SF-12 PCS and MCS scores, and WOMAC Pain score were comparable in THA and TKA, whereas THA patients reported slightly better self-rated health and poorer WOMAC Function score (Table 1).

**Table 1:** Baseline characteristics of the total hip arthroplasty (THA) and total knee arthroplasty (TKA) populations. Values are count (%) unless specified

	THA		TKA	
	Eligible	Included	Eligible	Included
Item	(n = 2,477)	(n = 1,772)	(n = 1,916)	(n = 1,323)
Female	1,375 (56)	982 (55)	1,250 (65)	843 (64)
Age at surgery **^a^**	68.4 (12.8)	68.8 (12.0)	70.2 (10.0)	70.9 (9.6)
Body mass index **^a^**	27.1 (5.3)	26.9 (5.2)	30.0 (5.6)	29.7 (5.4)
ASA score				
1	197 (8.0)	143 (8.1)	51 (2.7)	43 (3.3)
2	1,693 (68)	1,258 (71)	1,361 (71)	954 (72)
3–4	587 (24)	371 (21)	504 (26)	326 (25)
Number of comorbidities				
0	263 (11)	197 (11)	98 (5.1)	71 (5.4)
1–3	1,621 (65)	1,183 (67)	1,181 (62)	831 (63)
≥ 4	593 (24)	392 (22)	637 (33)	421 (32)
Smoking status				
Never	1,307 (53)	960 (54)	1,137 (60)	789 (60)
Former	648 (26)	473 (27)	486 (26)	348 (27)
Current	513 (21)	334 (19)	283 (15)	178 (14)
Missing	9 (	5 (	10 (	8 (
Education (years of schooling)				
≤ 8	729 (33)	536 (32)	700 (42)	501 (40)
9–12	704 (32)	525 (31)	523 (31)	398 (32)
≥ 13	770 (35)	616 (37)	453 (27)	340 (27)
Missing	274 (	95 (	240 (	84 (
Diagnosis				
Primary osteoarthritis	2,123 (86)	1,563 (88)	1,630 (85)	1,123 (85)
Secondary osteoarthritis	354 (14)	209 (12)	286 (15)	200 (15)
UCLA presurgery				
Low activity	1,594 (82)	1,176 (80)	1,236 (83)	886 (82)
Moderate activity	321 (16)	258 (18)	231 (16)	184 (17)
High activity	40 (2.0)	35 (2.4)	17 (1.1)	15 (1.4)
Missing	522 (	303 (	432 (	238 (
SF-12 MCS presurgery **^a^**	42.7 (11.2)	43.4 (11.2)	43.4 (11.5)	44.1 (11.5)
Missing	411 (	234 (	317 (	167 (
SF-12 PCS presurgery **^a^**	32.9 (7.2)	33.1 (7.2)	33.4 (7.1)	33.7 (7.1)
Missing	411 (	234 (	317 (	167 (
Self-rated health presurgery				
Poor–fair	407 (19)	278 (18)	359 (22)	227 (19)
Good	1,196 (57)	889 (57)	971 (60)	711 (61)
Very good–excellent	508 (24)	404 (26)	299 (18)	236 (20)
Missing	366 (	201 (	287 (	149 (
WOMAC Pain presurgery **^a^**	38.7 (18.1)	39.5 (18.1)	37.6 (17.1)	38.9 (17.0)
Missing	389 (	218 (	300 (	155 (
WOMAC Function presurgery **^a^**	39.7 (19.0)	40.6 (18.8)	42.2 (18.9)	43.7 (18.9)
Missing	420 (	241 (	330 (	175 (

a mean (SD)

**Figure 2:**
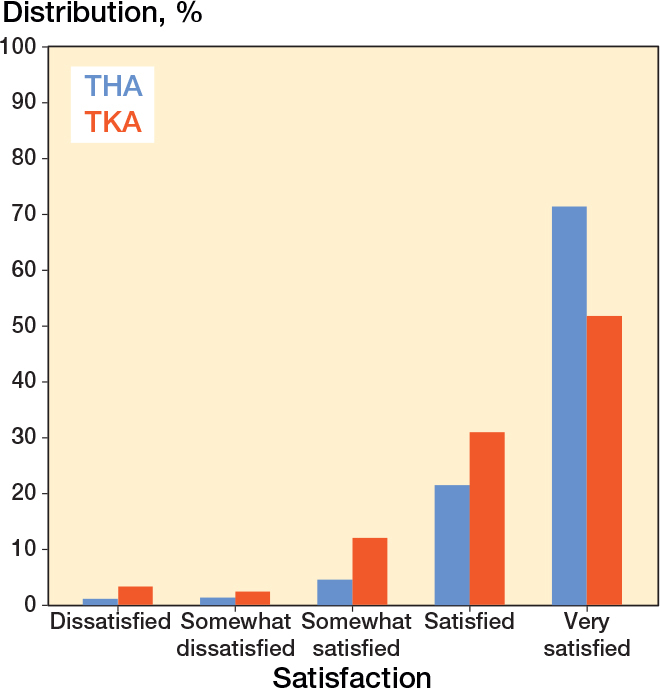
Distribution of patient satisfaction 1 year after total hip arthroplasty (THA) and total knee arthroplasty (TKA).

**Figure 3:**
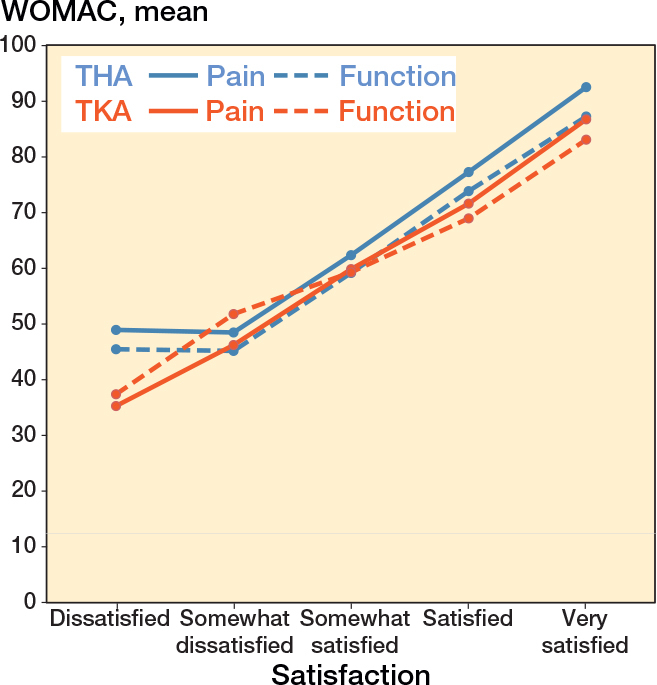
Mean WOMAC Pain & Function summary scores 1 year after surgery according to satisfaction level for total hip arthroplasty (THA) and total knee arthroplasty (TKA) populations.

1 year after surgery, THA patients reported higher satisfaction than TKA patients, with respectively 93% vs 82% being satisfied or very satisfied, and 7.1% vs 18% being somewhat satisfied, somewhat dissatisfied, or dissatisfied (Figure 2). In both THA and TKA populations, mean WOMAC Pain and Function summary scores increased with higher levels of satisfaction 1 year after surgery, highlighting the association between WOMAC summary scores and patient satisfaction at 1-year follow-up (Figure 3).

Both WOMAC Pain and Function summary scores showed strong individual associations with patient satisfaction in THA and TKA. Considering the mutually adjusted model including both summary scores, WOMAC Pain showed a much stronger association with patient satisfaction than WOMAC Function in both populations, although both remained significant (Table 2).

**Table 2:** Ordinal logistic regression models of WOMAC summary scores for the total hip arthroplasty (THA) and total knee arthroplasty (TKA) populations. Odds ratios are reported for a difference of 1 standard deviation

	THA		TKA	
	Univariable	Mutually adjusted ^a^	Univariable	Mutually adjusted ^a^
Score	Odds ratio (CI)	Odds ratio (CI)	Odds ratio (CI)	Odds ratio (CI)
WOMAC Pain	3.44 (3.07–3.87)	2.80 (2.40–3.29)	4.24 (3.72–4.86)	3.61 (2.96–4.44)
WOMAC Function	2.71 (2.43–3.02)	1.34 (1.14–1.56)	3.14 (2.78–3.55)	1.22 (1.01–1.47)

CI = 95% confidence interval.

a Mutually adjusted models include both WOMAC Pain & Function summary scores.

Similarly, all pain and function items showed strong individual associations with satisfaction in THA and TKA. Considering mutually adjusted models, which included either all pain or all function items, differences in the strength of associations were observed across items. Focusing on pain items, all were significantly associated with satisfaction in both populations, except “at night while in bed” in THA. Among significant items, “walking on the flat” and “going up or down stairs” had the strongest associations with patient satisfaction in both THA and TKA. Focusing on function items, 4 in THA—“ascending stairs,” “rising from sitting,” “walking on the flat,” and “putting on socks”—and 5 in TKA—“ascending stairs,” “rising from sitting,” “walking on the flat,” “getting in/out of car,” and “sitting”—were significantly associated with satisfaction. Among significant items, “walking on the flat,” “putting on socks,” and “ascending stairs” in THA and “walking on the flat,” “rising from sitting,” and “getting in/out of car” in TKA had the strongest associations. This suggests that pain while performing certain activities or limitations in performing specific tasks have a greater influence on patient satisfaction than others (Table 3). Furthermore, adjusting the models for sex, age at surgery, and BMI did not alter the findings (results not presented).

**Table 3:** Ordinal logistic regression models of WOMAC individual items for the total hip arthroplasty (THA) and total knee arthroplasty (TKA) populations

	THA		TKA	
	Univariable	Mutually adjusted ^a^	Univariable	Mutually adjusted ^a^
Item	Odds ratio (CI)	Odds ratio (CI)	Odds ratio (CI)	Odds ratio (CI)
Pain – walking on the flat	4.20 (3.65–4.85)	1.79 (1.45–2.22)	3.90 (3.41–4.48)	1.74 (1.43–2.11)
Pain – going up or down stairs	3.24 (2.88–3.65)	1.64 (1.37–1.96)	3.12 (2.78–3.51)	1.68 (1.44–1.97)
Pain – at night while in bed	2.81 (2.47–3.19)	1.14 (0.96–1.35)	2.74 (2.43–3.10)	1.29 (1.10–1.50)
Pain – rising or sitting on chair	3.25 (2.88–3.69)	1.34 (1.10–1.63)	2.87 (2.56–3.22)	1.21 (1.03–1.44)
Pain – standing upright	3.35 (2.95–3.80)	1.23 (1.00–1.51)	2.81 (2.51–3.17)	1.22 (1.04–1.44)
Function – ascending stairs	2.17 (1.97–2.39)	1.26 (1.08–1.46)	2.21 (2.00–2.45)	1.20 (1.04–1.40)
Function – rising from sitting	2.47 (2.20–2.77)	1.23 (1.02–1.48)	2.57 (2.30–2.88)	1.32 (1.11–1.58)
Function – walking on the flat	2.66 (2.36–3.00)	1.41 (1.17–1.68)	2.82 (2.47–3.18)	1.41 (1.18–1.69)
Function – getting in/out of car	2.40 (2.15–2.67)	1.20 (1.00–1.44)	2.54 (2.28–2.85)	1.31 (1.10–1.57)
Function – putting on socks	2.10 (1.91–2.32)	1.29 (1.12–1.48)	1.92 (1.75–2.12)	1.00 (0.87–1.15)
Function – rising from bed	2.44 (2.17–2.75)	1.05 (0.84–1.30)	2.47 (2.20–2.78)	1.00 (0.82–1.23)
Function – sitting	2.54 (2.24–2.88)	0.96 (0.77–1.21)	2.88 (2.53–3.28)	1.33 (1.08–1.63)

a Mutually adjusted models include either all WOMAC pain items or all WOMAC function items.

## Discussion

We aimed to examine whether certain functional abilities or pain during specific activities are associated more than other factors with satisfaction 1 year after THA and TKA. We identified differences in the contribution of patient outcomes to patient satisfaction with the result of THA and TKA interventions. Although both pain during activities and function at 1 year follow-up were significantly associated with patient satisfaction, pain had a greater influence, a finding consistent with prior research [15,21–23]. At the item level, pain while walking on flat ground and going up or down stairs demonstrated the strongest association with patient satisfaction in both THA and TKA. For functional activities, differences were highlighted between the 2 populations, with distinct activities contributing more strongly to satisfaction in each population. In THA, the ability to walk on flat ground, put on socks, and ascend stairs were the strongest contributors to satisfaction, whereas in TKA, walking on flat ground, rising from sitting, and getting in or out of a car had the greatest contribution.

At the 1-year follow-up, the proportion of satisfied to very satisfied patients in both THA and TKA populations is consistent with recent studies by Laigaard et al. [24,25]. Our findings provide novel evidence on the association between satisfaction and pain during specific activities after both THA and TKA. Regarding the association between satisfaction and functional activity limitations, results after THA are lacking. In contrast, after TKA, our findings are consistent with those of Nakahara et al., who investigated how difficulty in performing specific functional activities influenced patient satisfaction after TKA. They identified climbing up or down stairs, getting into or out of a car, moving laterally, and activities related to walking and standing as the most strongly associated with satisfaction [8]. Similarly, Noble et al. reported differences in the difficulty when performing specific activities between satisfied and dissatisfied patients after TKA, particularly in activities requiring knee function, such as climbing and descending stairs, getting up from sitting, walking on ramps or uneven surfaces, squatting, or gardening [11]. Although our study assessed a different set of items, similar functional activities, including walking on the flat, getting in or out of a car, ascending stairs, and rising from sitting, were identified as key contributors to patient satisfaction in the TKA population. Our findings also align with studies linking patient expectations to satisfaction. Lützner et al. identified improved knee ROM after TKA as one of the most important expectations for patients, with its fulfillment significantly contributing to patient satisfaction [9]. Similarly, Ghomrawi et al. showed that preoperative expectations of regaining full kneeling and leg-straightening abilities after TKA were amongst the strongest predictors of dissatisfaction [10]. These results underscore the importance of knee ROM after TKA, which is essential for performing several activities identified in our study, including ascending stairs, rising from sitting, and getting in or out of a car.

### Limitations

First, our analysis of the association between pain and function outcomes and patient satisfaction after primary elective THA and TKA was limited to 1-year follow-up. Therefore, the findings cannot be generalized to later follow-up points. We selected the 1-year follow-up point because it is the recommended time point for assessing PROs [5], given that most patients have reached full recovery by then, achieving maximal pain relief and functional improvement [26]. Beyond this period, only TKA patients have been reported to experience further improvements [27]. Second, various methods exist for measuring patient satisfaction, which may influence the result [6,7]. In this study, the measurement method employed has been recommended by the International Society of Arthroplasty Registries (ISAR) [5], ensuring alignment with international standards. Lastly, not all patients completed the WOMAC questionnaire and satisfaction question at 1-year follow-up. If the objective was to generalize numerical findings to the entire THA and TKA populations, missing data that does not follow a missing completely at random (MCAR) mechanism could potentially introduce bias. However, our goal was simply to compare various scale scores and items in their responsiveness to the effect of the interventions examined. Such comparison is useful even if the sample is not perfectly representative of the source population. Nevertheless, those outcomes were available for 74% of THAs and 72% of TKAs, both exceeding the 60% threshold considered sufficient [5], thereby supporting the use of a complete-case approach [28].

### Conclusion

Patient satisfaction was more strongly influenced by postoperative pain than by limitations in functional abilities after surgery. Pain while walking on flat ground and going up or down stairs were the most critical to patient satisfaction after both THA and TKA. These tasks are probably among the most common in daily life. Therefore, experiencing pain while performing them likely represents the greatest limitation and a major source of dissatisfaction. In contrast, the functional abilities associated with satisfaction differed between THA and TKA patients, reflecting the distinct biomechanical strains imposed on the hip or knee joints by these activities. In particular, difficulty putting on socks affected satisfaction only among THA patients, while difficulty getting in or out of a car impacted satisfaction among TKA patients only.

## Notes

Conceptualization: MR, TP, CB, HHM, DH, and AL. Methodology: MR, TP, and AL. Data curation: MR, CB, and AL. Formal analysis and visualization: MR, TP, and AL. Investigation and validation: MR, TP, CB, HHM, DH, and AL. Writing—original draft: MR, TP, and AL. Writing—review & editing: MR, TP, CB, HHM, DH, and AL. Supervision: TP, HHM, DH, and AL. All authors read and approved the final manuscript.

The authors thank all patients and orthopedic surgeons from the Division of Orthopedic Surgery and Musculoskeletal Trauma Care who have contributed information to the registry since 1996. They also express their gratitude to Carole Bandi, Flavia Renevey, and Lamia Blatter-Sellak for their invaluable assistance with data entry. 

Handling co-editors: Marianne Westberg and Robin Christensen

*Acta* thanks Mika Niemeläinen and Annette W-Dahl for help with peer review of this manuscript.

## Appendix

**Supplementary Table:** Ordinal logistic regression models of WOMAC summary scores for the total hip arthroplasty (THA) and total knee arthroplasty (TKA) populations. Comparison between change in scores from baseline to 1 year postsurgery (Δ) and scores at 1 year postsurgery. Odds ratios are reported for a difference of 1 standard deviation

	THA		TKA	
	Univariable	Mutually adjusted ^a^	Univariable	Mutually adjusted ^a^
Item	Odds ratio (CI)	Odds ratio (CI)	Odds ratio (CI)	Odds ratio (CI)
WOMAC Pain	3.44 (3.07–3.87)	3.18 (2.74–3.71)	4.24 (3.72–4.86)	4.02 (3.35–4.83)
Δ WOMAC Pain	2.34 (2.07–2.66)	1.15 (0.99–1.35)	2.67 (2.35–3.05)	1.14 (0.96–1.35)
WOMAC Function	2.71 (2.43–3.02)	2.43 (2.10–2.82)	3.14 (2.78–3.55)	3.03 (2.58–3.57)
Δ WOMAC Function	2.14 (1.89–2.43)	1.26 (1.08–1.46)	2.03 (1.80–2.30)	1.10 (0.95–1.28)

CI = 95% confidence interval.

a Mutually adjusted models include either WOMAC Pain and Δ WOMAC Pain or WOMAC Function and Δ WOMAC Function

## References

[1] Graham B, Green A, James M, Katz J, Swiontkowski M. Measuring patient satisfaction in orthopaedic surgery. J Bone Joint Surg Am 2015; 97(1): 80-4. doi: 10.2106/JBJS.N.00811. 25568398

[2] Hudak P L, McKeever P D, Wright J G. Understanding the meaning of satisfaction with treatment outcome. Med Care 2004; 42(8): 718-25. doi: 10.1097/01.mlr.0000132398.11342.a8. 15258473

[3] Lape E C, Hudak P, Davis A M, Katz J N. Body-self unity with a new hip or knee: understanding total joint replacement within an embodiment framework. ACR Open Rheumatol 2019; 1(2): 90-6. doi: 10.1002/acr2.1014. 31777785 PMC6857960

[4] Moore A, Eccleston C, Gooberman-Hill R. “It’s Not My Knee”: understanding ongoing pain and discomfort after total knee replacement through re-embodiment. Arthritis Care Res 2022; 74(6): 975-81. doi: 10.1002/acr.24534. PMC9311120 33290640

[5] Rolfson O, Bohm E, Franklin P, Lyman S, Denissen G, Dawson J, et al. Patient-Reported Outcome Measures Working Group of the International Society of Arthroplasty Registries. Patient-reported outcome measures in arthroplasty registries Report of the Patient-Reported Outcome Measures Working Group of the International Society of Arthroplasty Registries Part II. Recommendations for selection, administration, and analysis. Acta Orthop 2016; 87 (Suppl 1): 9-23. doi: 10.1080/17453674.2016.1181816. 27228230 PMC4937770

[6] Kahlenberg C A, Nwachukwu B U, McLawhorn A S, Cross M B, Cornell C N, Padgett D E. Patient satisfaction after total knee replacement: a systematic review. HSS J 2018; 14(2): 192-201. doi: 10.1007/s11420-018-9614-8. 29983663 PMC6031540

[7] Okafor L, Chen A F. Patient satisfaction and total hip arthroplasty: a review. Arthroplasty 2019; 1(1): 6. doi: 10.1186/s42836-019-0007-3. 35240763 PMC8787874

[8] Nakahara H, Okazaki K, Mizu-Uchi H, Hamai S, Tashiro Y, Matsuda S, et al. Correlations between patient satisfaction and ability to perform daily activities after total knee arthroplasty: why aren’t patients satisfied? J Orthop Sci 2015; 20(1): 87-92. doi: 10.1007/s00776-014-0671-7. 25366699

[9] Lützner C, Beyer F, David L, Lützner J. Fulfilment of patients’ mandatory expectations are crucial for satisfaction: a study amongst 352 patients after total knee arthroplasty (TKA). Knee Surg Sports Traumatol Arthrosc 2023; 31(9): 3755-64. doi: 10.1007/s00167-022-07301-y. 36740633 PMC10435619

[10] Ghomrawi H M K, Lee L Y Y, Nwachukwu B U, Jain D, Wright T, Padgett D, et al. Preoperative expectations associated with postoperative dissatisfaction after total knee arthroplasty: a cohort study. J Am Acad Orthop Surg 2020; 28(4): e145-e150. doi: 10.5435/JAAOS-D-18-00785. 31192886 PMC8362614

[11] Noble P C, Conditt M A, Cook K F, Mathis K B. The John Insall Award: Patient expectations affect satisfaction with total knee arthroplasty. Clin Orthop Relat Res 2006; 452: 35-43. doi: 10.1097/01.blo.0000238825.63648.1e. 16967035

[12] Lübbeke A, Baréa C, Miozzari H, Garavaglia G, Gonzalez A, Zingg M, et al. [Lessons learned from 25 years of an institutional hip and knee arthroplasty registry]. Rev Med Suisse 2021; 17(763): 2161-5. 34910401

[13] Lübbeke A, Hoogervorst L A, Marang-van de Mheen P J, Prentice H A, Rolfson O, Nelissen R G H H, et al., ISAR group. Arthroplasty registries at a glance: an initiative of the International Society of Arthroplasty Registries (ISAR) to facilitate access, understanding, and reporting of registry data from an international perspective. Acta Orthop 2025; 96: 116-26. doi: 10.2340/17453674.2024.42706. 39881617 PMC11760185

[14] Whitehouse S L, Lingard E A, Katz J N, Learmonth I D. Development and testing of a reduced WOMAC function scale. J Bone Joint Surg Br 2003; 85(5): 706-11. PMID: 12892194. 12892194

[15] Halawi M J, Jongbloed W, Baron S, Savoy L, Cote M P, Lieberman J R. Patient-reported outcome measures are not a valid proxy for patient satisfaction in total joint arthroplasty. J Arthroplasty 2020; 35(2): 335-9. doi: 10.1016/j.arth.2019.09.033. 31611162

[16] Tubach F, Dougados M, Falissard B, Baron G, Logeart I, Ravaud P. Feeling good rather than feeling better matters more to patients. Arthritis Rheum 2006; 55(4): 526-30. doi: 10.1002/art.22110. 16874795

[17] Statement on ASA Physical Status Classification System [Internet]. [accessed Jan 30, 2024]. Available from:https://journals.lww.com/anesthesiologyopen/fulltext/2026/01000/american_society_of_anesthesiologists_statement_on.2.aspx (cite as: American Society of Anesthesiologists Statement on ASA Physical Status Classification System. Anesthesiology Open 1(1):p e0002, January 2026. doi: 10.1097/ao9.0000000000000002)

[18] Zahiri C A, Schmalzried T P, Szuszczewicz E S, Amstutz H C. Assessing activity in joint replacement patients. J Arthroplasty 1998; 13(8): 890-5. doi: 10.1016/s0883-5403(98)90195-4. 9880181

[19] Ware J, Kosinski M, Keller S D. A 12-Item Short-Form Health Survey: construction of scales and preliminary tests of reliability and validity. Med Care 1996; 34(3): 220-3. doi: 10.1097/00005650-199603000-00003. 8628042

[20] Bilder C R, Loughin T M. Analysis of categorical data with R. 2nd ed. London: Chapman & Hall/CRC; 2015.

[21] Baker P N, van der Meulen J H, Lewsey J, Gregg P J, National Joint Registry for England and Wales. The role of pain and function in determining patient satisfaction after total knee replacement. Data from the National Joint Registry for England and Wales. J Bone Joint Surg Br 2007; 89(7): 893-900. doi: 10.1302/0301-620X.89B7.19091. 17673581

[22] Robertsson O, Dunbar M J. Patient satisfaction compared with general health and disease-specific questionnaires in knee arthroplasty patients. J Arthroplasty 2001; 16(4): 476-82. doi: 10.1054/arth.2001.22395a. 11402411

[23] Mannion A F, Kämpfen S, Munzinger U, Kramers-de Quervain I. The role of patient expectations in predicting outcome after total knee arthroplasty. Arthritis Res Ther 2009; 11(5): R139. doi: 10.1186/ar2811. 19772556 PMC2787271

[24] Laigaard J, Aljuboori S M, Nikolajsen L, Mathiesen O, Lunn T H, Lindberg-Larsen M, et al. Chronic pain after primary total and medial unicompartmental knee arthroplasty for osteoarthritis: a Danish nationwide cross-sectional survey. Acta Orthop 2025; 96: 814-21. doi: 10.2340/17453674.2025.44898. 41189424 PMC12559960

[25] Laigaard J, Aljuboori S M, Nikolajsen L, Mathiesen O, Lunn T H, Overgaard S. Chronic postsurgical pain after primary total hip arthroplasty for osteoarthritis: a nationwide cross-sectional survey study. J Arthroplasty 2025; S0883-5403(25)01249-5. doi: 10.1016/j.arth.2025.09.057. 41072560

[26] Galea V P, Rojanasopondist P, Ingelsrud L H, Rubash H E, Bragdon C, Huddleston III J I, et al. Longitudinal changes in patient-reported outcome measures following total hip arthroplasty and predictors of deterioration during follow-up: a seven-year prospective international multicentre study. Bone Joint J 2019; 101-B(7): 768-78. doi: 10.1302/0301-620X.101B7.BJJ-2018-1491.R1. 31256661

[27] Williams D P, Blakey C M, Hadfield S G, Murray D W, Price A J, Field R E. Long-term trends in the Oxford knee score following total knee replacement. Bone Joint J 2013; 95-B(1): 45-51. doi: 10.1302/0301-620X.95B1.28573. 23307672

[28] Christensen R, Ranstam J, Overgaard S, Wagner P. Guidelines for a structured manuscript: statistical methods and reporting in biomedical research journals. Acta Orthop 2023; 94: 243-9. doi: 10.2340/17453674.2023.11656. 37170796 PMC10176201

